# Cultural Adaptation and Validation of the CALMS Questionnaire in an Arabic-Speaking MS Population

**DOI:** 10.1093/arclin/acaf085

**Published:** 2025-09-27

**Authors:** Walid Al-Qerem, Sawsan Khdair, Dunia Basem, Anan Jarab, Judith Eberhardt

**Affiliations:** Department of Pharmacy, Faculty of Pharmacy, Al-Zaytoonah University of Jordan, Amman, Amman, Jordan; Department of Pharmacy, Faculty of Pharmacy, Al-Zaytoonah University of Jordan, Amman, Amman, Jordan; Department of Pharmacy, Faculty of Pharmacy, Al-Zaytoonah University of Jordan, Amman, Amman, Jordan; Department of Clinical Pharmacy, Faculty of Pharmacy, Jordan University of Science and Technology, Irbid, Irbid, Jordan; Department of Psychology, School of Social Sciences Humanities and Law, Teesside University, Borough Road, Middlesbrough, North Yorkshire, United Kingdom

**Keywords:** Multiple sclerosis, assessment, everyday functioning

## Abstract

Multiple sclerosis (MS) is a chronic neurological condition that often results in language and communication difficulties, significantly affecting patients’ quality of life. While several tools have been developed internationally to assess these impairments, few are validated for Arabic-speaking populations. This study aimed to translate, culturally adapt, and validate the Arabic version of the Communication and Language Assessment for Multiple Sclerosis (CALMS) questionnaire, designed to evaluate communication difficulties in individuals with MS. A cross-sectional study was conducted with 307 MS patients recruited from Al-Bashir Hospital in Amman, Jordan. Participants completed the Arabic CALMS (A-CALMS) via an online survey. Psychometric evaluation included Rasch analysis and Confirmatory Factor Analysis (CFA). Model fit, internal consistency (Cronbach’s alpha), person/item reliability, threshold ordering, and Differential Item Functioning (DIF) by sex were assessed. The partial credit model (sign) provided a better fit than the rating scale model. After reordering, all threshold parameters were properly ordered, and item/person fit statistics were acceptable. Internal consistency was excellent (α = 0.935); CFA supported a one-factor solution with good model fit (χ^2^/df = 1.82, RMSEA = 0.052, CFI = 0.987, TLI = 0.984). No significant DIF was found across sexes. A moderate positive correlation was observed between CALMS and MSIS-29 scores (*r* = 0.534, *p* < .001), supporting construct validity. The A-CALMS demonstrated strong psychometric properties and cultural relevance, making it a reliable, valid instrument for assessing communication and language difficulties in Arabic-speaking individuals with MS. Its use in clinical and research settings may enhance diagnostic precision and inform tailored interventions.

## INTRODUCTION

Multiple sclerosis (MS) is a chronic autoimmune disease that damages the central nervous system, causing demyelination and neuronal death, which underlie cognitive and physical deficits (Amato et al., 2019). Multiple sclerosis is known to affect patients’ physical abilities as well as cognitive functions such as language production and processing, impairing the ability to perform simple daily tasks and communicate with others (Rao et al., 1991). Despite the significant impact of physical impairment on the quality of life of people with MS (Vollset et al., 2024), studies have highlighted the substantial effect of word-finding difficulties and frequent grammatical errors on their quality of life and interpersonal communication (Amato et al., 2019). These difficulties can profoundly isolate people with MS by affecting social relationships, professional performance, and academic achievement (Chiaravalloti & DeLuca, 2008). Furthermore, the psychological stress of having trouble speaking and communicating can intensify feelings of depression and frustration, further affecting disease management (Sumowski et al., 2014). To assess speech impairments, recent studies have developed and validated tools to evaluate these challenges, such as the Communication and Language Assessment for persons with Multiple Sclerosis (CALMS) questionnaire (El-Wahsh et al., 2020), Cognitive Linguistic Quick Test (CLQT) (Mupawose & Broom, 2010), and Modified Fatigue Impact Scale (MFIS) (Farran et al., 2020). These tools are highly valuable for clinicians to accurately diagnose impairments, monitor disease progression, and select patient-specific rehabilitative interventions based on the severity of cognitive–linguistic deficits (Sumowski et al., 2014).

Despite their broad applicability, these assessment tools are primarily developed in English and are affected by cultural norms that may not align with those of Arabic-speaking populations (Benedict et al., 2003). Using these tools without cultural and linguistic adaptation risks misdiagnosing or overlooking key aspects of language and communication difficulties in Arabic-speaking individuals with MS (Fasfous & Daugherty, 2022).

This lack of culturally appropriate and validated tools in Arabic presents a significant obstacle to accurate clinical assessment, early intervention, and effective care planning. It also limits the scope and validity of research efforts aimed at understanding and addressing communication challenges in this population (Darwish et al., 2022). Given the growing recognition of MS-related communication challenges in Arabic-speaking populations, and the centrality of communication to quality of life, there is an urgent need for psychometrically sound instruments that are culturally relevant and accessible (Chiaravalloti & DeLuca, 2008). This study addresses that gap by translating, culturally adapting, and validating the Arabic version of the CALMS questionnaire. The aim is to provide a reliable and valid tool for identifying communication difficulties in Arabic-speaking individuals with MS, thereby enhancing both clinical practice and research in this area.

## MATERIALS AND METHODS

### Study Design and Participants

A cross-sectional study design was used to validate the Arabic version of CALMS in MS patients. Between January and May 2024, 307 individuals with MS were recruited from Al-Bashir Hospital in Amman, Jordan. Eligible participants were adults aged 18 or older who had been diagnosed with MS at least 1 year prior to enrollment. After obtaining patient contact details and relevant medical information, a trained researcher invited eligible individuals to take part in the study. The researcher explained the purpose of the study and emphasized that participation was entirely voluntary and would not affect patients’ medical care if they chose to withdraw. Participants who consented were sent a link to an online survey constructed using Google Forms, via the messaging application WhatsApp. The study was conducted in accordance with the ethical principles of the Declaration of Helsinki and was approved by the Al-Zaytoonah University of Jordan Research Ethics Committee (MOH/REC/2021/263).

### Study Instrument

Communication and Language Assessment for Multiple Sclerosis is a specialist tool designed to assess language and communication skills in individuals with neurological disorders such as multiple sclerosis. Because of the cognitive and motor impairments associated with MS, individuals often experience difficulties with speech production, comprehension, and pragmatic communication. The scale is designed to evaluate these aspects of language function (El-Wahsh et al., 2020). Communication and Language Assessment for Multiple Sclerosis includes a number of subtests that assess both verbal and non-verbal communication. These subtests evaluate fluency, articulation, comprehension, and the ability to use language effectively in social contexts. The scale has been used extensively in academic and clinical settings to assess the communication challenges faced by people with MS, providing valuable insight into how these impairments affect overall quality of life (El-Wahsh et al., 2020).

The questionnaire consists of 11 items that address various aspects of communication commonly affected in individuals with multiple sclerosis, including expressive and receptive language abilities, word-finding difficulties, attention and memory during conversations, and the emotional and social impact of communication problems. Participants responded to each item using a 4-point Likert scale ranging from “Never or rarely” to “Usually or always,” indicating the frequency of the issues they experience. Higher scores reflect a greater degree of communication difficulty. The CALMS questionnaire has demonstrated strong psychometric properties and provides valuable insight into how communication impairments affect patients’ daily functioning, interpersonal relationships, and overall quality of life (El-Wahsh et al., 2022). The patients were also invited to complete the Arabic Version of the Multiple Sclerosis Impact Scale (MSIS-29), which is a self-administered questionnaire developed to assess the impact of MS on individuals’ daily lives. It comprises 29 items divided into two subscales: 20 items evaluate the physical effects of MS, while the remaining 9 items focus on psychological aspects. The questionnaire has a four-point Likert scale ranging from “not at all” to “quite a bit/extremely.” Higher scores indicate a greater impact of the disease.

The study tool also included a demographic sheet that gathered information about patients’ age, gender, income status, education level, and employment and marital statuses.

### Data Collection and Sample Size

Phone numbers for 800 MS patients were obtained from Al-Bashir Hospital; however, approximately 80 patients either did not respond or had changed their contact information. A total of 321 patients were enrolled in the study. Of these, 20 participated in the pilot study, and the remaining patients completed the final questionnaire after receiving a link to a Google Form via WhatsApp.

The sample size for this study was determined based on commonly accepted guidelines for psychometric evaluations of self-report questionnaires. For exploratory or confirmatory analyses, a widely accepted rule of thumb is to recruit between 5 and 10 participants per questionnaire to ensure sufficient statistical power and stable estimates. Given that the CALMS questionnaire includes 11 items, the minimum recommended sample size ranged from 55 to 110 participants. To improve the reliability and generalizability of the findings, a larger sample size was targeted. Accordingly, the final sample of 307 participants exceeded the minimum requirements and allowed for robust analysis, including subgroup comparisons where appropriate.

A panel of experts, including two neurologists, an internal medicine physician, and a clinical pharmacist, evaluated the content validity of CALMS. The experts confirmed that the tool was comprehensive and addressed the various aspects of communication and language difficulties experienced by individuals with multiple sclerosis while maintaining clarity and simplicity in its language. The questionnaire underwent translation into Modern Standard Arabic in accordance with the Brislin model (Brislin, 1970), a widely adopted approach for maintaining both conceptual equivalence and cultural appropriateness in cross-linguistic research. Two professional translators independently drafted the instrument into Arabic, after which the two versions were carefully compared by the translators and research team. Through a collaborative review process, inconsistencies were identified and reconciled, resulting in a preliminary Arabic draft. This version was then subjected to back-translation by two separate bilingual translators unfamiliar with the original text. A comparative analysis of the back-translations, the source version, and the initial Arabic draft was conducted to resolve any remaining disparities and refine linguistic precision.

To assess face validity and ensure cultural and conceptual equivalence, a pilot study was conducted with 20 MS patients who were randomly selected from Al-Bashir Hospital and informed about the study’s purpose. Participants were invited to complete the tool and provide detailed feedback on the relevance, clarity, and ease of understanding of each item. Particular attention was paid to constructs that might be influenced by cultural or linguistic factors, such as “vague language use” and “difficulty organizing ideas,” to confirm that these concepts were meaningful and interpreted as intended within Arabic-speaking norms. During the pilot, an open discussion format was encouraged, allowing participants to elaborate on any ambiguities or alternative interpretations, especially regarding communication styles and everyday conversational behaviors. This approach was designed to capture any potential variation in how items might be understood, given differences in communication norms and expectations across Arabic-speaking populations. Feedback from participants, alongside input from translators and clinical specialists, was systematically reviewed and led to minor revisions to the tool, particularly in the phrasing of items with abstract or potentially culturally variable content. The pilot data were excluded from the main statistical analyses.

Additionally, Cronbach’s alpha was calculated to assess the internal consistency of CALMS. To further evaluate the tool’s ability to differentiate between varying levels of communication difficulties and to examine item-level characteristics, Rasch analysis was conducted. Rasch analysis is particularly useful for evaluating instruments designed to measure latent variables (Boone, 2016). For a comprehensive assessment, Wright maps, item fit, person fit, and item difficulty were examined to evaluate the tool’s psychometric quality. The model structure was confirmed by applying confirmatory factor analysis. In order to confirm CALMS predictability, CALMS scores we compared with the scores of the validated Arabic version of MSIS-29 (Al-Qerem et al., 2024).

### Statistical Analysis

A likelihood ratio test was performed to determine the most appropriate Rasch analysis approach (partial credit model [PCM] vs. rating scale model [RSM]). The partial credit model does not assume equal distances between response thresholds across all items and allows items to have varying numbers of response categories. In contrast, the rating scale model assumes uniform threshold distances. If the likelihood ratio test is significant (*p* < .05), the PCM is preferred (Ramp et al., 2009).

Rasch analysis was performed using Jamovi version 2.3.28, R (TAM) version 4.0-16, and eRm version 1.0-1. To evaluate the internal validity of CALMS, several Rasch model parameters were assessed. These included threshold ordering, unidimensionality, model fit, item fit, person fit, reliability, differential item functioning (DIF), and Q3 matrix.

CFA was used to assess the study data suitability to the suggested model suitability. The analysis was conducted using the lavaan package (version 0.6–17) in R, employing the WLSMV estimator (Weighted Least Squares Mean and Variance adjusted), which is appropriate for categorical ordinal data. Model fit was assessed using multiple indices, including χ^2^/df (minimum discrepancy), the Comparative Fit Index (CFI), the Standardized Root Mean Square Residual (SRMR), the Root Mean Square Error of Approximation (RMSEA), and the Tucker–Lewis Index (TLI). For model fit evaluation, the following criteria were applied: a χ^2^/df ratio less than 5 was considered acceptable (Alavi et al., 2020); RMSEA and SRMR values ≤0.08 indicated an acceptable fit; for the TLI, a value of 1 represented a perfect fit, while values approaching 1 suggested a very good fit (Widaman & Helm, 2023); and the CFI ranged from 0 to 1, with 1 indicating a perfect fit and values ≥0.9 reflecting an excellent fit (Hooper et al., 2007). Furthermore, factor loadings produced by CFA were also assessed. The internal consistency of the scale was evaluated using Cronbach’s alpha (Tavakol & Dennick, 2011).

## RESULTS

### Demographics

The demographic data indicate that the sample was predominantly female (68.4%), with a median age of 35 years (IQR: 28–44), suggesting a relatively young adult population. A substantial proportion of participants reported a monthly income below 500 Jordanian dinars (73.9%) and were unemployed (68.4%), reflecting potential socioeconomic vulnerability. In terms of educational attainment, 45% of respondents held a bachelor’s degree or higher, while 32.9% had completed high school or less, indicating a moderate level of educational diversity. The majority of participants were married (60.9%) (Table 1).

**Table 1:** Sociodemographic characteristics of the sample (*N* = 307)

Variable		Median (IQR) or frequency (%)
Age		35 (28–44)
Sex	Female	210 (68.4%)
	Male	97 (31.6%)
Income status (Jordanian Dinars per month)	<500	227 (73.9%)
	≥500	80 (26.1%)
Education level	High school or less	101 (32.9%)
	Diploma	68 (22.1%)
	Bachelor’s degree or higher	138 (45%)
Employment status	Unemployed	210 (68.4%)
	Employed	97 (31.6%)
Marital status	Not married	120 (39.1%)
	Married	187 (60.9%)

### Patients’ Responses to the CALMS Items

As shown in Table 2, the highest frequency for the response “Never or rarely” was recorded for the item “Use a lot of vague or empty words such as ‘you know what I mean’ instead of the right word” (31.9%), while the lowest was for “Find it difficult to concentrate enough to understand what is being said?” (18.9%). For the “Sometimes” category, the highest response rate was again observed for the use of vague or empty words (47.6%), whereas the lowest was for “Find it difficult to put ideas together in a logical way?” (34.9%). Under the “Often/Usually or always” category, the highest frequency was for “Find it difficult to remember information on the tip of your tongue?” (42.4%), while the lowest was again for the use of vague or empty words (20.5%).

**Table 2:** Reordered Frequencies (%) of the Communication and Language Assessment questionnaire for persons with Multiple Sclerosis (CALMS)

Item	Never or rarely	Sometimes	Often/Usually or always
Have difficulty thinking of the particular word you want?	69 (22.5%)	127 (41.4%)	111 (36.1%)
Have difficulty remembering your train of thought as you are speaking?	68 (22.1%)	119 (38.8%)	120 (39.1%)
Use a lot of vague or empty words such as “you know what I mean” instead of the right word?	98 (31.9%)	146 (47.6%)	63 (20.5%)
Find it difficult to remember information on the tip of your tongue?	59 (19.2%)	118 (38.4%)	130 (42.4%)
Find it difficult to convey precisely what you mean?	69 (22.5%)	120 (39.1%)	118 (38.4%)
Leave out important details?	83 (27%)	126 (41%)	98 (31.9%)
Find it difficult to concentrate enough to understand what is being said?	58 (18.9%)	123 (40.1%)	126 (41%)
Find it difficult to remember recent conversations?	69 (22.5%)	110 (35.8%)	128 (41.7%)
Find it difficult to keep track of the main details of conversations?	88 (28.7%)	118 (38.4%)	101 (32.9%)
Find it difficult to put ideas together in a logical way?	80 (26.1%)	107 (34.9%)	120 (39.1%)
Hesitate, pause, or repeat yourself?	80 (26.1%)	122 (39.7%)	105 (34.2%)

### Tool Validation

The likelihood ratio test for CALMS was significant, supporting the use of a PCM (*p* < .001). The Rasch rating scale model showed poorer data-model fit; therefore, the PCM for polytomous responses was selected. The Wright map for CALMS before reordering indicated some threshold disordering in CALMS 3, CALMS 6, and CALMS. Several rescoring options were tested. However, all disordered thresholds were resolved by reducing the original 4-point response scale to a 3-point scale. This was achieved by merging the two upper response categories (“Often” and “Usually or always”), as shown in Table 2. Moreover, CFA was conducted to evaluate and validate the questionnaire. The model demonstrated acceptable fit, with χ^2^/df = 1.82, RMSEA = 0.052 (90% CI: 0.033–0.070), SRMR = 0.041, CFI = 0.987, and TLI = 0.984. All indices met recommended thresholds, indicating that the proposed one-factor solution was appropriate for the current sample and all factor loadings were above 0.6 (Table 3). The internal consistency of the scale was excellent, as indicated by a Cronbach’s alpha of 0.935 across the 11 items, suggesting high reliability. While the original 4-point response scale yielded a Cronbach’s alpha of 0.91 and model fit indices of χ^2^/df = 3.11, RMSEA = 0.083 (90% CI: 0.067–0.099), SRMR = 0.053, CFI = 0.962, and TLI = 0.953, with factor loadings ranging from 0.53 to 0.85, these results indicate that the proposed adjustment improved model fit.

**Table 3:** Factor loadings, fit statistics, item measures, and tau thresholds for CALMS Scale items after reordering

Item	Factor loadings	Infit	Outfit	Measure	tau parameters	
					1	2
CALMS 1	0.63 (0)	1.53	1.71	−0.52	−1.84	−1.05
CALMS 2	0.86 (0.09)	0.84	0.87	−0.65	−1.97	−1.31
CALMS 3	0.68 (0.09)	1.29	1.48	0.45	−0.86	0.91
CALMS 4	0.9 (0.09)	0.74	0.67	−0.90	−2.22	−1.80
CALMS 5	0.9 (0.09)	0.80	0.72	−0.61	−1.93	−1.23
CALMS 6	0.89 (0.09)	0.79	0.72	−0.18	−1.50	−0.36
CALMS 7	0.85 (0.09)	0.94	0.88	−0.86	−2.18	−1.72
CALMS 8	0.84 (0.09)	0.97	0.89	−0.74	−2.06	−1.49
CALMS 9	0.88 (0.09)	0.88	0.80	−0.15	−1.47	−0.31
CALMS 10	0.9 (0.09)	0.88	0.77	−0.50	−1.81	−1.00
CALMS 11	0.71 (0.09)	1.32	1.41	−0.31	−1.62	−0.61

**Fig. 1:**
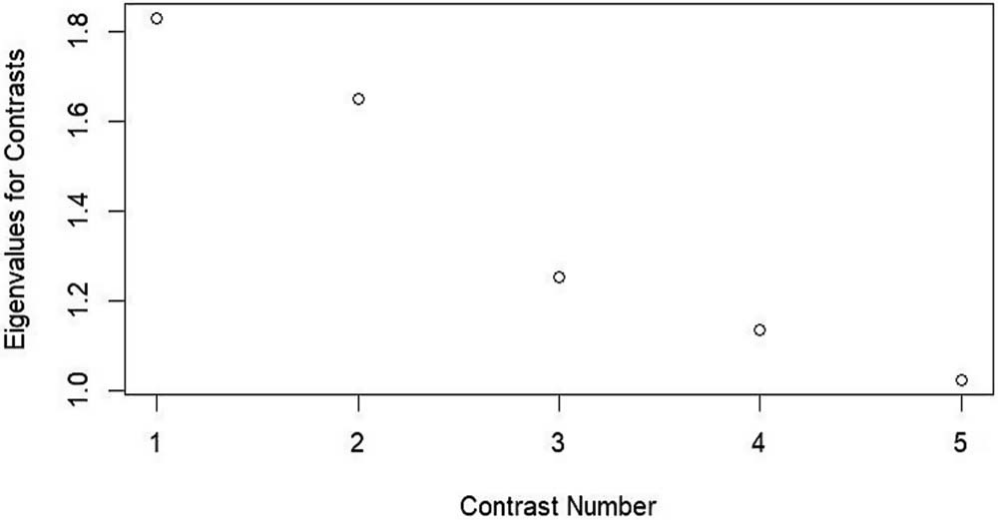
Contrasts from principal component analysis of the residuals.

The Rasch analysis confirmed the suitability of the model with all Infit and outfit MSQ values within the acceptable range (0.6–1.4) except for CALMS 1, which had an infit of 1.53 and an outfit of 1.71. Regarding item difficulty, estimates ranged from −0.90 for CALMS 4 to 0.45 for CALMS 3, covering a reasonable span of the latent trait and reflecting the scale’s ability to differentiate across varying levels of communicative difficulty. An examination of person fit statistics revealed that the vast majority of respondents fell within acceptable fit ranges, with both infit and outfit *t*-values clustering around the expected mean. Only a few individuals exceeded the conventional threshold of ±2, indicating isolated instances of unexpected response behaviour. Similarly, standardized residual analysis showed that only 12 participants (fewer than 4% of the sample, *N* = 307) fell outside the acceptable ±2 range. Principal Component Analysis of Residuals (PCAR) was used to evaluate unidimensionality by ensuring the first contrast eigenvalue was <2 and the analysis of the unidimensionality of the model as the eigenvalue for the first contrast recorded at approximately 1.9 (Fig. 1).

**Fig. 2:**
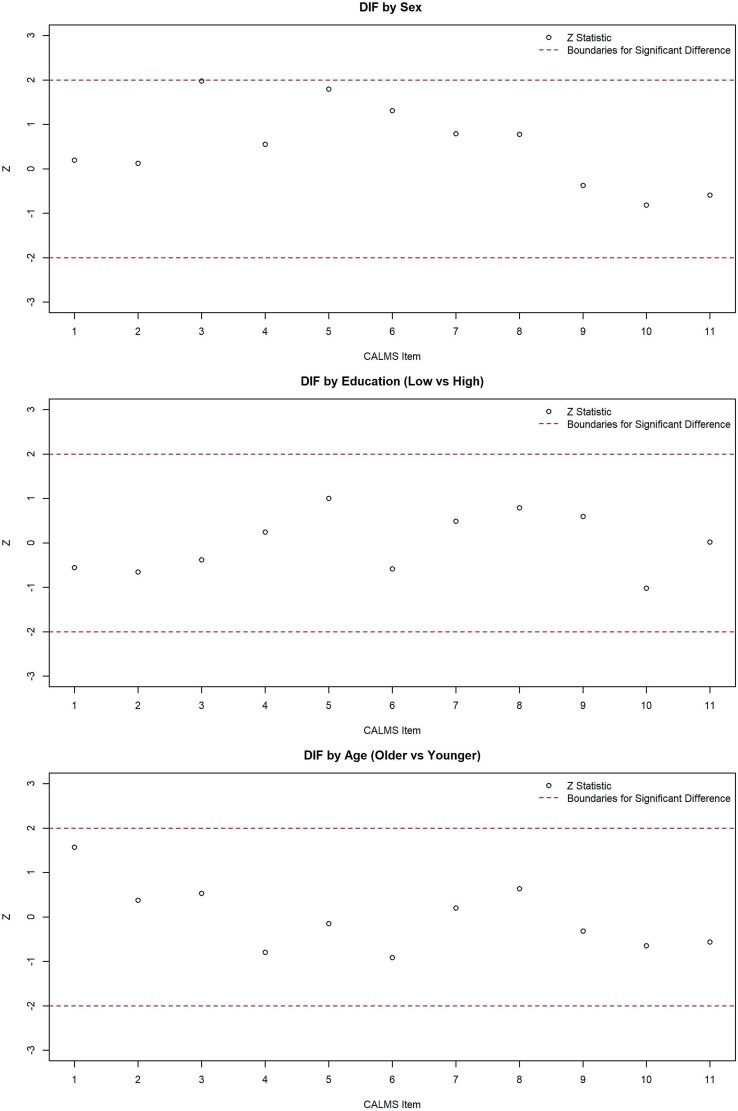
Differential Item Functioning (DIF) analysis between item comparisons between sexes, age groups, and education level.

A review of the Q3 residual correlation matrix showed that most item pairs had low residual correlations, indicating good local independence across the scale. However, the highest residual correlation was found between CALMS 1 and CALMS 9 (*r* = 0.355), which exceeds 0.30 but remains less than 0.5. DIF analysis indicated no significant item-level bias by sex, age group (above the median of 35 vs. below the median), and education level (Bachelor’s degree or higher vs. diploma or less). All Z statistics were within the acceptable ±2 range, supporting measurement invariance and confirming that the scale functioned equivalently for all subgroups of respondents (Fig. 2).

**Fig. 3:**
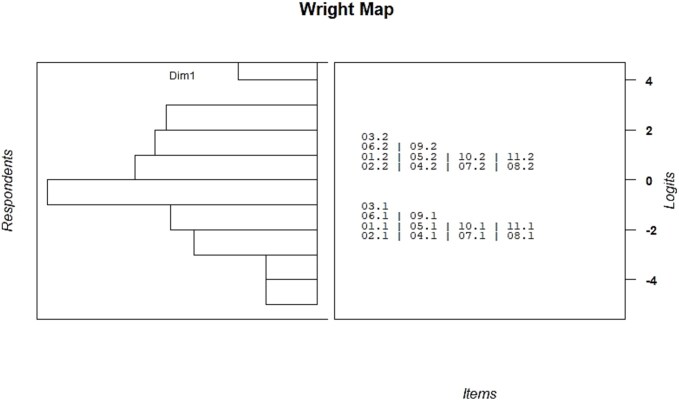
Wright map: the distribution of person abilities (on the left) and item difficulties (on the right).

The Wright map displays the distribution of person abilities (on the left) and item difficulties (on the right) along a common logit scale (Fig. 3). Overall, the spread of item difficulties appears reasonably well matched to the distribution of respondent abilities, although there is a noticeable clustering of respondents toward the lower end of the scale. The items cover a good range of logit values, with some clustering around the center, suggesting adequate measurement targeting for the majority of participants.

A Pearson correlation analysis revealed a statistically significant moderate positive correlation between the total CALMS score and the MSIS-29 total score (*r* = 0.534, *p* < .001), indicating that greater language and communication difficulties are associated with higher levels of MS-related impact.

## DISCUSSION

This study translated, culturally adapted, and validated the A-CALMS questionnaire. The results provide strong evidence for the reliability, validity, and cross-cultural applicability of CALMS among Arabic-speaking individuals with MS.

The internal consistency of the A-CALMS was excellent (Cronbach’s α = 0.935), far exceeding the conventional threshold of 0.70 for acceptable reliability. This high alpha suggests that the items are measuring a coherent underlying construct and that the scale produces stable and consistent scores across respondents. These results are comparable to those reported for the original English version of the CALMS, which also demonstrated strong internal consistency in a clinical MS sample (El-Wahsh et al., 2020). Importantly, this consistency was achieved despite cultural and linguistic differences, highlighting the success of the translation and adaptation process in maintaining conceptual equivalence. Nevertheless, the high Cronbach’s α may also suggest some overlap or redundancy among items. However, given the focus of this study on validation rather than instrument revision, and considering the brevity of the CALMS, all items were retained to preserve content validity.

In addition to Cronbach’s alpha, the high person reliability coefficient indicated that the A-CALMS can reliably differentiate between individuals with different levels of communication difficulty. This is particularly important in the context of MS, where subtle variations in cognitive–linguistic function may significantly affect everyday activities and social participation (Chiaravalloti & DeLuca, 2008; El-Wahsh et al., 2022). A reliable instrument ensures that changes over time, whether due to disease progression or therapeutic intervention, can be meaningfully interpreted rather than dismissed as measurement error.

These findings affirm the scale’s utility for both cross-sectional assessment as well as longitudinal monitoring in clinical settings, where repeated evaluation is often necessary to track progression or treatment response. Given the historical lack of validated communication assessment tools in Arabic for neurological populations, the strong reliability of the A-CALMS marks a significant contribution to clinical neuropsychology in the region.

Rasch analysis supported the structural validity of the A-CALMS, confirming that the scale items functioned well in measuring the intended construct of communication difficulty. The use of the PCM was appropriate for capturing variations in how respondents interacted with the response categories. During analysis, it became clear that some participants struggled to distinguish between certain response options, prompting a revision of the original response scale for selected items. This adjustment improved the functioning of the response categories and enhanced the clarity of measurement.

The majority of items showed good fit with the underlying trait of communicative difficulty, indicating that the scale items were both meaningful and interpretable for respondents. However, one item, related to the use of vague or empty words, showed signs of poor fit, suggesting it may not align as strongly with the overall construct. This echoes findings from earlier studies on communication assessment tools in MS, where items involving subtle metacognitive behaviors have shown weaker psychometric performance (El-Wahsh et al., 2020, 2022). It may reflect variability in self-awareness or cultural communication norms, which can influence how such behaviors are interpreted and reported. While this item was retained to maintain content coverage, its performance suggests it may benefit from revision in future versions of the tool.

The item difficulty estimates indicated that the A-CALMS captured a suitable range of communicative challenges, spanning from relatively simple to more complex language-related tasks. This suggests that the scale is capable of detecting variability in communication abilities across individuals with MS. While the Wright map revealed a clustering of respondents with lower estimated difficulty levels, the analysis of total scores showed that only 4.2% of participants scored at the minimum possible level. This finding demonstrates that the scale does not exhibit a significant floor effect at the scale level and is able to differentiate even among higher-functioning individuals. The observed distribution may reflect characteristics of the sample, which consisted predominantly of young adults, many with higher education and likely earlier disease stages. At the item level, several questions did display a substantial proportion of lowest-category responses (up to 31.9%), suggesting some clustering among participants with very mild difficulties. Nonetheless, the overall sensitivity of the CALMS appears adequate for capturing a broad spectrum of communicative challenges. In future applications, particularly in more heterogeneous or less impaired populations, the addition of items targeting higher-level pragmatic or discourse skills could further enhance the tool’s ability to detect mild impairments.

The structural validity of the A-CALMS was reinforced by confirmatory factor analysis, which supported a unidimensional model. This finding replicates the original validation study (El-Wahsh et al., 2020) and indicates that the scale items consistently reflect a single latent construct, namely, the impact of MS on communication and language functioning. That this factor structure held in an Arabic-speaking sample is important, as it confirms that the construct is conceptually and psychometrically meaningful across cultural contexts. This is a key step in validating instruments for use in linguistically diverse settings, especially when applied to conditions like MS that manifest with complex, multidimensional symptoms.

Evidence for construct validity was also strong. The A-CALMS total score was moderately correlated with the Arabic version of the MSIS-29, a well-established measure of MS-related physical and psychological impact (Al-Qerem et al., 2024). This relationship aligns with prior research showing that cognitive and communication impairments meaningfully contribute to patients’ perceptions of disease burden and can be just as disruptive to daily life as physical limitations (Farran et al., 2020). These findings point to the clinical relevance of assessing language difficulties in MS, which are frequently overshadowed by more visible symptoms but play a critical role in social participation, employment, and emotional well-being.

Finally, the absence of DIF by sex, education level, or age group suggests that the A-CALMS performs equivalently for all demographic subgroups. This supports the scale’s measurement invariance and ensures that comparisons across subgroups are meaningful. This finding is consistent with other validated Arabic-language MS measures such as BICAMS and MSIS-29, which have also demonstrated psychometric equivalence across demographic subgroups (Al-Qerem et al., 2024; Darwish et al., 2022). Taken together, these results strengthen confidence in the tool’s fairness and generalizability, supporting its use in both clinical assessment and epidemiological research involving mixed-gender samples in Arabic-speaking contexts. However, future work could also examine measurement invariance across additional demographic variables, such as rural versus urban residence, as these data were not available for the current sample.

Overall, the findings of the present study highlight the robustness of the A-CALMS and its potential to address the longstanding gap in linguistically and culturally appropriate communication assessments for Arabic-speaking MS populations. In the Middle East and North Africa region (Heydarpour et al., 2015; Sahraian et al., 2010), where MS prevalence is and healthcare systems are increasingly emphasizing patient-centered care (Alkhaibari et al., 2023), validated tools such as CALMS are essential for both clinical management and research. Its use may support more targeted interventions, improved patient–provider communication, and greater understanding of how communication challenges affect quality of life and disease outcomes.

The modification of the response scale from four to three categories in the A-CALMS resulted in improved model fit and slightly higher internal consistency, without altering the overall structure or interpretability of the instrument. The psychometric properties observed for the Arabic version following this adjustment remain highly comparable to those reported in validation studies of the original English CALMS using the four-point scale. For example, indices of internal consistency and unidimensional factor structure in both versions fall within the same range, and the pattern of item–factor loadings is similar (El-Wahsh et al., 2020). Importantly, score interpretation remains aligned: Higher total scores continue to reflect greater communication difficulty, and the clinical meaning of individual item responses is preserved. However, users should be aware that total scores derived from the three-point version may exhibit a slightly different distributional range compared to the four-point version. While normative values may need to be re-established for specific populations using the three-point format, the core interpretive guidelines and clinical thresholds developed for the CALMS remain fundamentally applicable, supporting comparability of findings across studies and languages.

Based on the improved psychometric performance observed with the three-point version in the present study, we recommend that the three-point response format be adopted as the standard for future applications of the A-CALMS in Arabic-speaking contexts, both clinically and in research. This version not only enhances measurement clarity and category functioning but may also reduce cognitive demands on respondents, which is particularly relevant for individuals with neurological or cognitive impairment. Nevertheless, for cross-linguistic or multinational research, we advise that investigators either harmonize response formats across language versions or provide clear documentation of any differences in scoring protocols to ensure comparability of results. More broadly, the findings of the present study suggest that the simplification of response categories should be carefully considered in other language adaptations of the CALMS or similar tools, especially in populations where cognitive load or response ambiguity may affect data quality. Future research in other languages and clinical populations may help clarify whether category reduction consistently enhances psychometric properties and respondent experience.

### Strengths, Limitations, and Future Directions

A key strength of this study is its rigorous, theory-informed approach to validation. Using Rasch analysis and CFA, the study provides a comprehensive evaluation of the psychometric properties of the A-CALMS. These complementary methods offered a detailed assessment of the scale’s internal structure, item functioning, and overall reliability. The translation and cultural adaptation process was an additional strength, incorporating input from clinical experts and end users to ensure the tool remained conceptually accurate and accessible for Arabic-speaking individuals with MS.

The relatively large and clinically relevant sample, drawn from a major neurological treatment center, enhances the generalizability of the findings to similar Arabic-speaking populations. In addition, the use of the Arabic version of the MSIS-29, a widely validated external measure, to assess construct validity strengthens confidence in the scale’s ability to capture meaningful aspects of communication difficulty that are linked to the broader impact of MS.

However, several limitations should be acknowledged. First, the sample was recruited from a single urban hospital in Jordan. Although the participants were demographically diverse, the findings may not reflect the full range of experiences found across Arabic-speaking populations in other regions or healthcare systems. For example, individuals in rural areas or those without access to digital devices may have been underrepresented due to the online survey format. Furthermore, although the A-CALMS was developed using Modern Standard Arabic to maximize broad accessibility, it is possible that comprehension or interpretation of questionnaire items may differ among individuals with limited education, rural residents, or speakers of strong regional dialects. Local linguistic and cultural norms could influence how communication behaviors are expressed or understood, potentially affecting response validity in some groups. Future validation efforts should aim to include participants from multiple centers and settings, as well as diverse educational and linguistic backgrounds, to improve external validity and ensure the instrument’s appropriateness across the broader Arabic-speaking world.

Second, one item on the scale showed evidence of misfit, which may reflect challenges in how certain concepts are interpreted. In particular, language behaviors that rely on self-awareness, such as using vague or empty words, may be more context-sensitive or culturally variable (McGee, 2018). Qualitative work could help refine these items by exploring how Arabic-speaking individuals with MS perceive and describe their communication challenges, ensuring the content reflects culturally relevant expressions and real-life language use.

A further limitation relates to the relatively high education level observed in the sample. While this may have supported comprehension of the tool, it may also have increased participants’ self-awareness of milder communication difficulties, which could have either improved the accuracy of self-reporting or, conversely, led to underreporting due to heightened stigma or social desirability. This dynamic may have contributed to the clustering of lower scores observed in the sample, and future studies should explore how educational background influences both response patterns and the validity of self-reported communication challenges. Future research should conduct additional validation studies in other Arabic-speaking countries and among populations with a broader range of educational backgrounds, rural and urban contexts, and regional dialects. These efforts will help confirm the psychometric properties of the A-CALMS and may guide necessary adaptations to enhance its cultural and linguistic applicability across diverse settings.

Stigma surrounding cognitive and communication difficulties in MS may contribute to social desirability bias, potentially leading some participants to underreport the extent of their impairments. To help mitigate this risk, the researcher emphasized to all participants that their responses would remain strictly confidential, participation was voluntary, and no identifying information would be linked to their survey data. This assurance of confidentiality was intended to promote honest and accurate reporting. Nevertheless, self-report measures are inherently susceptible to bias, and the risk of underreporting cannot be fully excluded. To further strengthen the validity in future research, information from clinician assessments or caregiver reports can be assessed to provide additional context and help verify the accuracy of self-reported communication challenges.

Looking ahead, the A-CALMS has potential applications beyond initial screening. It could be used in longitudinal studies to monitor changes over time or in clinical trials evaluating the effectiveness of communication-focused interventions. The development of companion versions for caregivers or healthcare providers could also enhance the tool’s utility by capturing different perspectives on communication function. In addition, adaptation for use in other neurological conditions, such as stroke or traumatic brain injury, could expand its relevance in Arabic-speaking healthcare systems. However, it is important to pilot test the instrument in these groups to ensure that all items are relevant and comprehensible. Certain items, particularly those addressing higher-order language and executive functions, may require modification or supplementation to reflect the distinct communication profiles observed in these conditions.

## CONCLUSION

This study provides strong evidence for the reliability and validity of the Arabic version of the CALMS questionnaire as a tool for assessing communication and language difficulties in individuals with multiple sclerosis. Through a rigorous process of translation, cultural adaptation, and psychometric evaluation, the scale was shown to have excellent internal consistency, sound structural validity, and measurement invariance across gender. These findings support its use in clinical and research settings across Arabic-speaking populations, where appropriate assessment tools have been lacking. While further research is needed to evaluate the scale’s performance in more diverse populations and over time, the A-CALMS represents a valuable contribution to the assessment of cognitive-linguistic functioning in MS and offers a foundation for more culturally responsive care.
